# Treatment-Related Cardiovascular Outcomes in Patients with Symptomatic Subclavian Artery Stenosis

**DOI:** 10.7759/cureus.1262

**Published:** 2017-05-19

**Authors:** Narendranath Epperla, Fan Ye, Amr Idris, Adeeb Sakkalaek, Hong Liang, Po-Huang Chyou, Richard A Dart, Joseph Mazza, Steven Yale

**Affiliations:** 1 Hematology and Oncology, Medical College of Wisconsin; 2 GME Internal Medicine Residency Program, North Florida Regional Medical Center; 3 Biomedical Informatics Research Center, Marshfield Clinic Research Foundation; 4 Center for Human Genetics, Marshfield Clinic Research Foundation; 5 Department of Clinical Research, Marshfield Clinic Research Foundation; 6 Internal Medicine, University of Central Florida College of Medicine

**Keywords:** subclavian artery stenosis, atherosclerosis, percutaneous trans-luminal angioplasty, bypass surgery, stent, adverse cardiovascular event, antiplatelet, pharmacotherapy, intervention, patient outcome assessment

## Abstract

**Background:**

Subclavian artery stenosis (SAS) is narrowing of the subclavian artery most commonly caused by atherosclerosis. It serves as a marker for cerebrovascular and myocardial ischemic events.

**Methods:**

A retrospective cohort study was conducted to determine the association of treatment via combination therapy (antiplatelet drug plus either by-pass surgery or percutaneous transluminal angioplasty (PTA) with or without stent implantation) versus antiplatelet drug therapy alone on cardiovascular events and all-cause mortality in Marshfield Clinic patients diagnosed with symptomatic SAS from January 1, 1995 to December 31, 2009.

**Results:**

Of the total 2153 cases, 100 patients were identified as eligible to be included in the study. Of these 100 patients that met inclusion criteria, 30 underwent combination therapy while 70 were managed only with drug treatment. A median length of follow-up was 8.45 years. Adverse cardiovascular events occurred in 5/30 (17%) of combination therapy patients compared to 28/70 (40%) of antiplatelet drug therapy only patients (p = 0.0355). Accordingly, all-cause mortality was higher (47%) in the antiplatelet drug therapy only group than the combination therapy group (13%) [hazard ratio = 3.45, p = 0.0218].

**Conclusions:**

Preliminary findings in this pilot data set suggest that combination therapy (medications plus either surgical or interventional repair) of subclavian artery stenosis is associated with less cardiovascular adverse events and higher survival rates. However, prospective randomized studies with larger number of patients are needed to validate these findings.

## Introduction

Subclavian artery stenosis (SAS) is a rare cause of extracranial artery disease with an incidence between 0.6% and 6%. SAS is usually identified in males between 40 and 60 years of age and typically involves proximal portion of left subclavian artery [1-4]. SAS is most commonly caused by atherosclerotic occlusive vascular disease, but has also been described in patients with stenosis caused by fibromuscular dysplasia [5], Takayasu syndrome [2], radiation exposure [6], post-traumatic compression syndrome [7], and polymyalgia rheumatica [1]. SAS may also occur in those who have undergone coronary artery bypass surgery with a left internal mammary artery graft with ensuing angina or myocardial ischemia [8]. Consequences of SAS include chronic arterial insufficiency leading to vertebra-basilar insufficiency or arm claudication.

Diagnosis of hemodynamically significant subclavian artery stenosis requires identifying asymmetrical systolic blood pressure measurements in the upper extremities and ultrasonographic/angiographic evidence of subclavian stenosis and retrograde flow. The threshold for upper extremity systolic blood pressure difference (SBPD) for diagnosis of clinically significant SAS is uncertain. Inter-arm measurements ranging from 10 to 20 mm Hg have been assessed with varying levels of specificity and sensitivity [4, 9-10]. A recent meta-analysis reported that an SBPD of 10–15 mm Hg is associated with an increased risk of vascular disease and cardiovascular mortality [11]. It has been suggested that patients requiring coronary artery bypass grafting (CABG) with an SBPD of at least 10 mm Hg or evidence of peripheral vascular disease (PVD) undergo selective arteriography screening at the time of coronary catheterization [12]. In either case, using this threshold for screening underestimates the true prevalence of SAS and carries a sensitivity of approximately 65% [10].

Guidelines for performing non-invasive imaging modalities, such as duplex ultrasonography or computed tomography (CT) or magnetic resonance (MR) angiography of the subclavian artery, to screen for SAS have not been verified. Some authors recommend routine non-invasive angiographic screening for SAS prior to CABG [13], even in the absence of signs and symptoms [14], whereas others recommend selective subclavian angiography only in the presence of symptoms or physical examination findings [4, 15].

Treatment of subclavian artery stenosis is driven by the presence of symptoms (arm claudication, dizziness, syncope, dysarthria, diplopia) or cardiac ischemia. Management typically includes conservative treatments with medications to reduce the risk of cardiovascular events such as stroke and myocardial infarction and if applicable, invasive therapy including surgical or interventional procedures. With respect to revascularization and achievement of anterograde flow in the vertebral, there are various approaches including open surgery/endarterectomy, extrathoracic bypass, and endovascular stenting. Less invasive strategies including extrathoracic bypass and percutaneous stenting have largely replaced traditional open endarterectomy, particularly in patients who are at increased risk for surgery [16-17].

Percutaneous transluminal angioplasty (PTA) with stenting is considered the treatment of choice [18], although a recent Cochrane review suggests that there is no direct evidence that angioplasty with stent placement provides an advantage over angioplasty alone [19]. PTA has been consistently shown to have favorable outcomes and short- and long-term vessel patency [3], particularly in patients with symptomatic disease [18]. Complications, such as restenosis and poor outcomes, arise primarily in patients with multiple stent placements, low stent diameter, and lower SBPD [20]. A 50% failure rate has been reported with endovascular intervention in patients with occlusion/stenosis [21].

Several studies have assessed the association between SAS and other vascular disorders and cardiovascular risk factors [9, 12, 22], but none to date has examined the role that noninvasive combined invasive intervention for SAS may play in predicting the cardiovascular risk and mortality in the future. Therefore, this study sought to assess the incidence of cardiovascular events and all-cause mortality in symptomatic SAS patients that underwent combination therapy (drug plus invasive intervention including PTA with/without stent placement or bypass surgery) versus drug therapy only. We hypothesized that patients with symptomatic SAS who did not receive an invasive intervention were at increased risk for mortality and major cardiovascular events compared to those who underwent both noninvasive (i.e., medications only) and invasive intervention.

## Materials and methods

The work has been carried out in accordance with The Code of Ethics of the World Medical Association (Declaration of Helsinki) for experiments involving humans; Uniform Requirements for manuscripts submitted to Biomedical journals.

### The study population

A retrospective cohort study of patients seen within the Marshfield Clinic Health System, Marshfield, WI was conducted from January 1, 1995 to December 31, 2009. Following institutional review board approval with a waiver of informed consent, patients were initially identified through an electronic query of the Marshfield Clinic electronic medical record system using International Classification of Disease, Version 9 (ICD9) codes 447.1 (stricture of artery) and 435.2 (subclavian artery syndrome). Patients included in this study were at least 18 years of age and diagnosed clinically and radiographically as having symptomatic SAS. Demographic and clinical data collected for all subjects included gender, race, body mass index, smoking history, hypertension, dyslipidemia, and comorbid conditions defined using the Charlson comorbidity index [23].

### Definition of terms

Subclavian artery stenosis was defined as occlusion in the first part of the subclavian artery causing stenosis which was diagnosed through either: asymmetric blood pressure detected in the upper extremities (interarm SBPD of at least 15 mm Hg), doppler ultrasonographic or angiographic evidence of >60% subclavian stenosis, or 100% occlusion, or symptoms compatible with SAS. Patients with SAS were then divided into two groups based on the presence or absence of symptoms. Patients who met the case definition of SAS but did not have any symptoms were defined as asymptomatic SAS. Symptomatic SAS was defined as patients who met the case definition described above with any of the following symptoms: arm claudication (reproducible upper extremity pain upon exertion), arm weakness or fatigue, arm rest pain; clinical stigmata of vertebra-basilar insufficiency (vertigo, ataxia or balance disturbances, amaurosis fugax); or leg claudication (reproducible lower extremity pain upon exertion in a patient with previous axillary-femoral graft).

### Study variables

Differences in the occurrence of cardiovascular events, including acute myocardial infarction, ischemic stroke, coronary arterial occlusion, and all-cause mortality were evaluated in patients who underwent an invasive intervention (PTA with or without stent or bypass surgery [axillo-axillary, carotid subclavian, or subclavian-axillary bypass]) plus antiplatelet drug therapy versus antiplatelet drug therapy alone. Antiplatelet agents were administrated at the time of referenced diagnosis.

Outcome variables were manually extracted and included the occurrence of cardiovascular events (i.e., PVD, carotid artery disease, recurrent angina, acute coronary syndrome, transient ischemic attack, and stroke) assessed at years one through five and six or more from the referenced date of diagnosis.

### Statistical analysis

The percentages of the following variables were compared between the combination therapy group (n = 30) and drug therapy only group (n = 70) using Chi-square test or Fisher’s Exact test when appropriate: demographics (gender, dyslipidemia, hypertension, race, smoking status, subclavian artery bruit, peripheral pulses) and outcomes (PVD, carotid artery disease, acute coronary syndrome, recurrent angina, stroke, transient ischemic attack, subclavian steal, and any cardiovascular event). In addition, the medians of the following variables were compared between the above-mentioned two groups using nonparametric Wilcoxon Rank Sums Test: Charlson score, body mass index, diastolic and systolic blood pressures, total cholesterol, high-density lipoprotein, and triglycerides. Kaplan-Meier survival curves were plotted to compare the difference between the two treatment groups; statistical significance of Hazard Ratio (HR) for treatment allocation was tested using the Cox proportional hazard regression model by adjusting age, gender and Charlson score. A p-value of less than 0.05 was considered statistically significant. All data analyses were carried out using a commercially available statistical software package, SAS (version 9.4).

## Results

**Figure 1:**
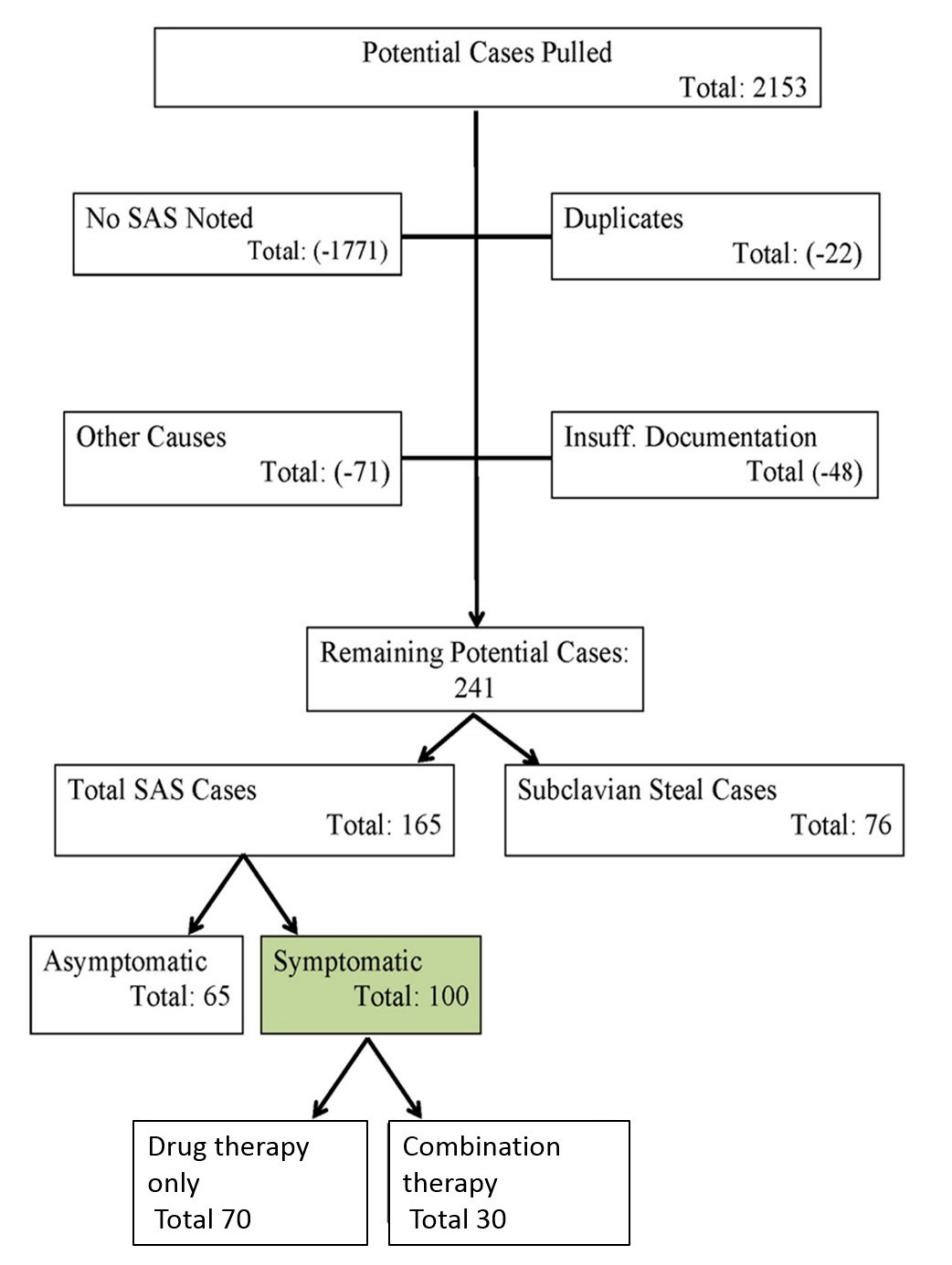
Flow diagram of search results. Patients were electronically screened by ICD9 code and manually verified as shown among the patient records in Marshfield between January 1, 1995 and December 31, 2009. SAS: Subclavian artery stenosis.

Of 2153 initial cases of SAS identified electronically, a total of 165 patients were manually adjudicated as having SAS (Figure 1). One of the major causes for exclusion (n = 1771) was the lack of SAS diagnosis, since electronic query using the aforementioned ICD9 code also identified vascular occlusion/stenosis involving other vessels, such as carotid artery stenosis or renal artery stenosis. Of the 165 patients, 65 were asymptomatic and excluded from further analysis. The remaining 100 symptomatic SAS cases were divided based on which intervention they received. Invasive interventions were performed in 30 of 100 symptomatic SAS patients.

In the combination therapy group (n = 30), 19 patients had PTA with stent placement (n = 3 for right PTA/stent and n = 16 for left PTA/stent) while 11 patients had bypass surgery (n = 1 for subclavian-axillary bypass, n = 2 for axillo-axillary bypass, and n = 8 for carotid-subclavian bypass). In the non-invasive intervention group (i.e., drug treatment only), patients were treated with antiplatelet agents (e.g., aspirin (81-325 mg), clopidogrel). A median length of follow-up was 8.45 years. Demographic factors and comorbidities were largely similar between the combination therapy and drug therapy groups, with a few notable exceptions (Table 1).

**Table 1:** Demographic and clinical characteristics by treatment group for 100 patients with symptomatic subclavian artery stenosis (SAS).

	Drug therapy only		Combination therapy		
Variable	Total	n (%) or median (range)	Total	n (%) or median (range)	p-value
Gender	70		30		0.5291^c^
Female		42 (60.0%)		20 (66.7%)	
Male		28 (40.0%)		10 (33.3%)	
Age	70	70.6 (42.4-90)	30	62.1 (40.8-90)	0.1493^b^
Race	70		30		0.2347^a^
White		60 (85.7%)		28 (93.3%)	
Other		1 (1.4%)		1 (3.3%)	
Not documented		9 (12.9%)		1 (3.3%)	
Smoking Status	70		30		
Current		20 (28.6%)		15 (50.0%)	0.0395^c^
Past/never		49 (70.0%)		14 (46.7%)	
Not documented		1 (1.4%)		1 (3.3%)	
Dyslipidemia	70	50 (71.4%)	30	18 (60.0%)	0.2616^c^
Hypertension	70	48 (68.6%)	30	19 (63.3%)	0.6097^c^
Physical Findings					
Bruit	67	50 (74.6%)	23	16 (69.6%)	0.6358^c^
Palpable peripheral pulses	65	28 (43.1%)	25	22 (88.0%)	*0.0001*^a^
Comorbidities					
Charlson score	70	1.0 (0.0–4.0)	30	0.0 (0.0–4.0)	0.2334^b^
BMI (kg/m^2^)	53	27.6 (19.0–45.9)	25	27.1 (19.7–36.7)	0.7529^b^
DBP (mm Hg)	61	70.0 (42.0–96.0)	28	71.0 (58.0–96.0)	0.9269^b^
SBP (mm Hg)	61	130.0 (66.0–186.0)	28	137.0 (102.0–180.0)	0.3312^b^
Cholesterol (mg/dl)	64	192.0 (75.0–304.0)	27	199.0 (111.0–330.0)	0.9689^b^
HDL (mg/dl)	63	46.0 (23.0–89.0)	26	41.0 (26.0–100.0)	0.7256^b^
Triglycerides (mg/dl)	63	143.0 (59.0–325.0)	26	136.0 (46.0–660.0)	0.9749^b^
Symptoms					
Arm claudication	70	18 (25.7%)	30	14 (46.7%)	0.0396^c^
Vertigo/dizzy	70	14 (20.0%)	30	5 (16.7%)	0.6970^c^
Arm/hand pain	70	21 (30.0%)	30	8 (26.7%)	0.7364^c^
Syncope	70	4 (5.7%)	30	2 (6.7%)	*1.0000*^a^
Amaurosis fugax	70	8 (11.4%)	30	1 (3.3%)	*0.2717*^a^
Headache	70	6 (8.6%)	30	2 (6.7%)	*1.0000*^a^
Ataxia, unsteadiness	70	6 (8.6%)	30	1 (3.3%)	*0.6711*^a^
Other	70	25 (35.7%)	30	13 (43.3%)	0.4719^c^
Location of SAS Right Left	70 70	29(41.4%) 56(80.0%)	30 30	11(36.7%) 22(73.3%)	*0.8241*^a^ *0.5988*^a^
SAS: Subclavian artery stenosis; BMI: Body mass index; DBP: Diastolic blood pressure; SBP: Systolic blood pressure; HDL: High-density lipoprotein. Significant p-values are italicized. ^a^ p-value derived from Fisher’s Exact test. ^b^ p-value derived from Wilcoxon Rank Sums test. ^c^ p-value derived from Chi-square test.					

**Figure 2:**
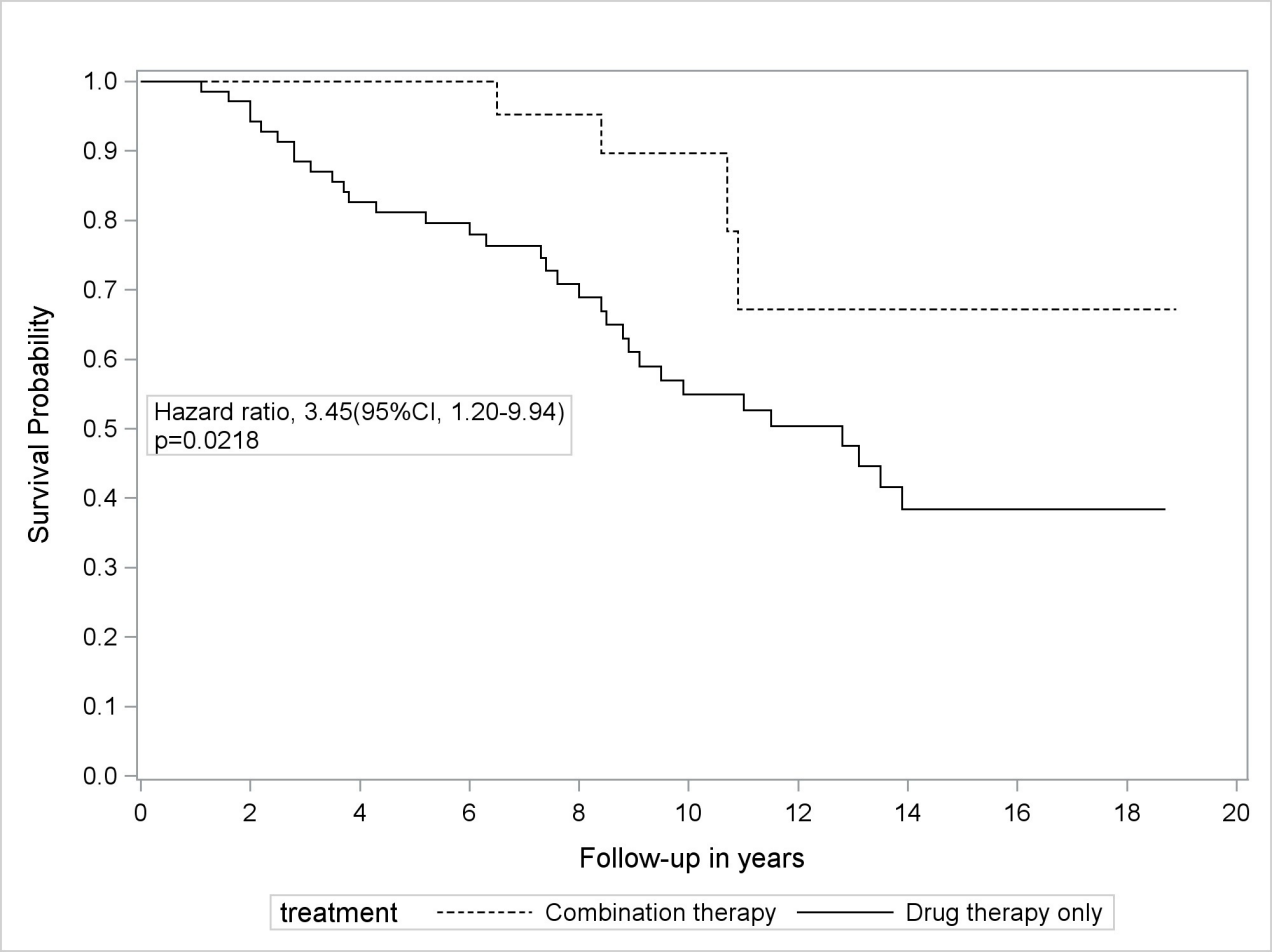
Kaplan-Meier survival curves for symptomatic subclavian artery stenosis (SAS) patients according to their intervention status drug therapy only (n = 70) versus combination therapy (n = 30) group.

Table 1 shows that invasive intervention was more likely to be performed in the patients with current smoking status (50% at combination therapy group vs. 29% at drug therapy only group, p = 0.0395), the patients with palpable peripheral pulses (88% at combination therapy group vs. 43% at drug therapy only group, p = 0.0001), and the patients with arm claudication symptom (47% at combination therapy group vs. 26% at drug therapy only group, p = 0.0396). There was no statistically significant difference in treatment assignment in younger patients (median age of 62.1 at combination therapy group vs. 70.6 at drug therapy only group, p = 0.1493). Patients with symptomatic SAS who did not receive PTA or surgical intervention were more likely to experience a cardiovascular event (40% vs. 17%, p = 0.0355) and death (hazard ratio = 3.45, p = 0.0218) compared to those who did undergo an invasive intervention (Table 2, Figure 2). In addition, PTA application was associated with the lower mortality rate (10.5% for PTA vs 47.1% for drug therapy only; p < 0.05).

**Table 2:** Primary outcomes by treatment group for 100 patients (70 no intervention vs 30 intervention) with symptomatic subclavian artery stenosis (SAS). Combination therapy refers to by-pass surgery or percutaneous transluminal angioplasty (PTA) with or without stent implantation plus drug therapy versus drug therapy only.

Variable	Drug therapy only; Total (70), n (%)	Combination therapy; Total (30), n (%)	p-value
CE			*0.0355*^a^
Yes	28 (40.0%)	5 (16.7%)	
No	42 (60.0%)	25 (83.3%)	
Overall survival			
Death	33 (47.1%)	4 (13.3%)	*0.0218*^ b^
Censored hazard ratio (95% CI)^ c^	37 (52.9%) 3.45 (1.198, 9.941)	26 (86.7%)	
CE categories^d^			
Peripheral vascular disease	6 (8.6%)	3 (10.0%)	0.9999^a^
Carotid artery disease	12 (17.1%)	1 (3.3%)	0.1012^a^
Acute coronary syndrome	5 (7.1%)	0 (0%)	0.3184^a^
Recurrent angina	5 (7.1%)	1 (3.3%)	0.6654^a^
Transient ischemic attack	6 (8.6%)	0 (0%)	0.1744^a^
Stroke	1 (1.4%)	1 (3.3%)	0.5121^a^
SAS: Subclavian artery stenosis; CE: Cardiovascular event. Significant p-values are italicized. ^a^ p-value derived from Fisher’s Exact test. ^b^ p-value and ^c^ Hazard ratio (95%CI) derived from Cox proportional hazards model adjusted for age, gender and Charlson score. ^d ^Cardiovascular Events (Some patients belonged to more than one category).			

The difference in cardiovascular event rate could not be attributed to a specific type of event and was only evident when all events were considered cumulatively due to small sample size. Table 3 depicts the use of aspirin and/or clopidogrel among symptomatic SAS patients by the status of intervention.

**Table 3:** Use of aspirin and/or clopidogrel by treatment group for 100 patients with symptomatic subclavian artery stenosis (SAS).

	Drug therapy only	Combination therapy	
Variable	n (%)	n (%)	p-value^a^
Aspirin			1.000
Yes	22 (31.4%)	9 (30.0%)	
No	48 (68.6%)	21 (70.0%)	
Clopidogrel			*0.0053*
Yes	62 (88.6%)	19 (63.3%)	
No	8 (11.4%)	11 (36.7%)	
Significant p-values are italicized. ^a ^p-values derived from Fisher’s Exact test.			

The use of clopidogrel was statistically significantly higher in the drug therapy only group (89%) compared with combination therapy group (63%, p = 0.0053). Of note, all the patients with symptomatic SAS in both groups were on a statin (since this is a retrospective study, no certain data for duration could be obtained from the records). Restenosis occurred in 4/19 (21%) patients with symptomatic SAS in the intervention group who underwent PTA with stent placement within five years of intervention, a rate similar to that identified in other studies [24-26]. The median duration for restenosis (n = 4) was noted to be 45 months in our study (Table 4).

**Table 4:** Characteristics of subclavian artery stenosis (SAS) patients with restenosis.

Patient	Side	Number of stents	Stent restenosis duration	Medications
1	R	1	6 mo	ASA + Clopidogrel
2	R	1	24 mo	ASA + Clopidogrel
3	L	1	60 mo	ASA + Clopidogrel
4	L	1	42 mo	ASA + Clopidogrel
			Median duration: 45 mo	
R: Right; L: Left; mo: Months; ASA: Acetylsalicylic acid.				

## Discussion

To our knowledge, this is the first study to explore the clinical outcomes between endovascular and surgical intervention combined with antiplatelet medical therapy with antiplatelet treatment alone. Our results showed that combination therapy was associated with a lower risk of adverse cardiovascular events and longer survival rates.

SAS is an occlusive vascular disease most commonly caused by atherosclerosis. Most patients with SAS are asymptomatic [3]. However, when stenosis of the subclavian or innominate artery is significant enough to cause gradient-driven reversal of normal flow with symptoms referable to the vertebra-basilar, coronary systems, and/or the upper extremities treatment should be considered. Numerous recent studies have established the association of SAS with the presence of subclinical atherosclerotic disease [10-12], thus highlighting the potential role for the presence of SAS to be used as a marker for identifying patients at high risk of developing future cardiovascular events.

PTA has become the intervention of choice in patients with symptomatic SAS, although pharmacotherapy alone is also commonly employed. PTA with stenting has been shown to have a high procedural success rate and is efficacious in alleviating symptoms with a cumulative patency rate of 89% at 40 months [25]. Given the association of SAS with atherosclerosis and the availability of a successful treatment modality for symptomatic SAS, this study explored the implications of SAS intervention on cardiovascular events and all-cause mortality.

The majority of studies evaluating surgical procedures or PTA in patients with SAS have shown favorable vessel patency rates extending beyond 10 years [24, 27-28]. PTA with or without stenting is currently considered the treatment of choice for patients with SAS [18], but whether stenting alters procedural efficacy is currently unknown [19]. Angioplasty of the subclavian artery has been shown consistently to have favorable outcomes and favorable short and long-term patency results particularly in patients with stenosis [3], and is recommended especially in symptomatic patients [18]. The development of endovascular therapy has fundamentally changed the management of subclavian steal syndrome patients. It should be noted that a 50% failure rate with endovascular intervention has been reported in patients with occlusion compared to those with stenosis [21]. Factors that are more likely to lead to restenosis and poorer outcome include implantation of more than one stent, low stent diameter, and systolic blood pressure differences in the upper extremities (after the stent placement) [20]. While questions regarding treatment efficacy are important, studies often fail to report on patient outcomes in those who did not undergo invasive intervention or received drug therapy alone, which also represents an unresolved or unanswered clinical question.

In the present study, only 30% of patients with symptomatic SAS underwent surgical or PTA intervention. The study revealed that comorbid conditions could not be accounted for receiving an invasive intervention as there was no difference identified between the groups based on disease severity, while current smoking status, palpable peripheral pulses, and arm claudication symptom may be accounted for receiving an invasive intervention. Little information is available regarding physician judgment regarding perceived versus actual risk in patients deemed to be at high risk for an interventional procedure. Patients with both prohibitive surgical risk and/or unfavorable anatomy for percutaneous treatment may prevent them from receiving invasive procedures. Studies in patients undergoing cardiothoracic surgery, carotid endarterectomy, and arterial surgery have shown that using indices of frailty may be useful in assessing post-operative risk and mortality [29]. Further studies should explore these perceptions and preferences from the patient and physician perspectives with respect to SAS to better understand treatment options.

Although there is no similar trial, the combination of drug therapy with invasive interventions from our study has shown some promising results. Medical therapy with antiplatelet therapy is currently recommended, regardless the degree of “steal” in SAS patients. Patients benefit from aggressive medical therapy primarily targeting reducing the risk for sub­clavian arterial atherosclerosis. One possibility is antiplatelet therapy is associated with improved graft patency [30], which may further reduce cardiovascular event after invasive interventions in the combination therapy group.

One of the major strengths of our study was that patients were identified in a clinical setting and not from a referral population, were racially homogenous and all had symptomatic disease. However, there are several limitations of this study. First, this is a retrospective study and hence is prone to bias and incomplete information, such as the reason why certain patients received combination therapy vs. drug therapy only. Second, the sample size is small, although one should note that this is a relatively rare disease, and we have chosen only patients with symptomatic SAS, which makes it even more difficult to have large numbers. Other limitations include predominant Caucasian patient population residing in the rural area where the study was carried out. Also, physician characteristics such as years of medical practice, number of different physicians performing these procedures and procedural performance are other factors that may have influenced outcomes. Data was not available regarding these variables to draw any conclusions and should be evaluated in future studies.

## Conclusions

Preliminary findings in our relatively small data cohort of patients suggest that invasive intervention of subclavian artery stenosis combined with drug therapy is associated with less cardiovascular adverse events and higher survival rates. However, further prospective randomized studies with larger number of patients are needed to validate these findings.

In the population examined here, drug combined with either PTA or surgical management of symptomatic SAS was associated with a significant reduction in major adverse cardiovascular outcomes and improved all-cause mortality. In addition, PTA application was associated with lower mortality. It is very possible that the combination therapy group could receive more clinical care and education to teach them to modify their lifestyles. To our knowledge, this is the first study to establish such findings in relation to the decision for invasive interventional treatment of symptomatic SAS. In the context of the limitations described above, there are several points of interest. First, interventions with PTA or surgery should be strongly considered in all symptomatic SAS patients, barring any patient contraindication, given the association with improved cardiovascular morbidities and overall survival. Second, these findings warrant further investigation in the form of a prospective randomized clinical trial with a larger sample size to better understand patient, physician, and environmental factors that influence medical decision making as there is strong evidence of a beneficial risk/benefit short- and long-term profile in patients undergoing intervention associated with improved cardiovascular morbidity and all-cause mortality.

## References

[1] Ghetie D, Rudinskaya A, Dietzek A (2010). Polymyalgia rheumatica with bilateral subclavian artery stenosis. Am J Med.

[2] Gowda AR, Gowda RM, Gowda MR (2004). Takayasu arteritis of subclavian artery in a Caucasian. Int J Cardiol.

[3] Labropoulos N, Nandivada P, Bekelis K (2010). Prevalence and impact of the subclavian steal syndrome. Ann Surg.

[4] Osborn LA, Vernon SM, Reynolds B (2002). Screening for subclavian artery stenosis in patients who are candidates for coronary bypass surgery. Catheter Cardiovasc Interv.

[5] Luscher TF, Lie JT, Stanson AW (1987). Arterial fibromuscular dysplasia. Mayo Clin Proc.

[6] Smith E, Magee B (2003). Arm pain due to subclavian artery stenosis after radiotherapy for recurrent breast cancer. Clin Oncol (R Coll Radiol).

[7] Lee EB, Seo KS (2003). Acute symptomatic traumatic subclavian steal syndrome: case report. J Trauma.

[8] Sadek MM, Ravindran A, Marcuzzi DW (2008). Complete occlusion of the proximal subclavian artery post-CABG: presentation and treatment. Can J Cardiol.

[9] English JA, Carell ES, Guidera SA (2001). Angiographic prevalence and clinical predictors of left subclavian stenosis in patients undergoing diagnostic cardiac catheterization. Catheter Cardiovasc Interv.

[10] Shadman R, Criqui MH, Bundens WP (2004). Subclavian artery stenosis: prevalence, risk factors, and association with cardiovascular diseases. J Am Coll Cardiol.

[11] Aboyans V, Criqui MH, McDermott MM (2007). The vital prognosis of subclavian stenosis. J Am Coll Cardiol.

[12] Aboyans V, Kamineni A, Allison MA (2010). The epidemiology of subclavian stenosis and its association with markers of subclinical atherosclerosis: the Multi-Ethnic Study of Atherosclerosis (MESA). Atherosclerosis.

[13] Feit A, Reddy CV, Cowley C (1992). Internal mammary artery angiography should be a routine component of diagnostic coronary angiography. Cathet Cardiovasc Diagn.

[14] Rigatelli G, Rigatelli G (2005). Screening angiography of supraaortic vessels performed by invasive cardiologists at the time of cardiac catheterization: indications and results. Int J Cardiovasc Imaging.

[15] Olsen CO, Dunton RF, Maggs PR (1988). Review of coronary-subclavian steal following internal mammary artery-coronary artery bypass surgery. Ann Thorac Surg.

[16] Cua B, Mamdani N, Halpin D (2017). Review of coronary subclavian steal syndrome. J Cardiol.

[17] Salman R, Hornsby J, Wright LJ (2016). Treatment of subclavian artery stenosis: a case series. Int J Surg Case Rep.

[18] Henry M, Henry I, Polydorou A (2007). Percutaneous transluminal angioplasty of the subclavian arteries. Int Angiol.

[19] Iared W, Mourao JE, Puchnick A (2014). Angioplasty versus stenting for subclavian artery stenosis. Cochrane Database Syst Rev.

[20] Przewlocki T, Kablak-Ziembicka A, Pieniazek P (2006). Determinants of immediate and long-term results of subclavian and innominate artery angioplasty. Catheter Cardiovasc Interv.

[21] Linni K, Ugurluoglu A, Mader N (2008). Endovascular management versus surgery for proximal subclavian artery lesions. Ann Vasc Surg.

[22] Kimura A, Hashimoto J, Watabe D (2004). Patient characteristics and factors associated with inter-arm difference of blood pressure measurements in a general population in Ohasama, Japan. J Hypertens.

[23] Charlson ME, Pompei P, Ales KL (1987). A new method of classifying prognostic comorbidity in longitudinal studies: development and validation. J Chronic Dis.

[24] Babic S, Sagic D, Radak D (2012). Initial and long-term results of endovascular therapy for chronic total occlusion of the subclavian artery. Cardiovasc Intervent Radiol.

[25] Bates MC, Broce M, Lavigne PS (2004). Subclavian artery stenting: factors influencing long-term outcome. Catheter Cardiovasc Interv.

[26] Reivich M, Holling HE, Roberts B (1961). Reversal of blood flow through the vertebral artery and its effect on cerebral circulation. N Engl J Med.

[27] Duran M, Grotemeyer D, Danch MA (2015). Subclavian carotid transposition: immediate and long-term outcomes of 126 surgical reconstructions. Ann Vasc Surg.

[28] Mousa AY, AbuRahma AF, Bozzay J (2015). Anatomic and clinical predictors of reintervention after subclavian artery stenting. J Vasc Surg.

[29] Sepehri A, Beggs T, Hassan A (2014). The impact of frailty on outcomes after cardiac surgery: a systematic review. J Thorac Cardiovasc Surg.

[30] Stein PD, Dalen JE, Goldman S (2001). Antithrombotic therapy in patients with saphenous vein and internal mammary artery bypass grafts. Chest.

